# The impact of self-esteem on the preferential processing of self-related information: Electrophysiological correlates of explicit self vs. other evaluation

**DOI:** 10.1371/journal.pone.0200604

**Published:** 2018-07-16

**Authors:** Maria M. Nowicka, Michał J. Wójcik, Ilona Kotlewska, Michał Bola, Anna Nowicka

**Affiliations:** 1 Laboratory of Psychophysiology, Nencki Institute of Experimental Biology, Polish Academy of Sciences, Warsaw, Poland; 2 Faculty of Humanities, Nicolaus Copernicus University, Torun, Poland; 3 Department of Psychological and Brain Sciences, Dartmouth College, Hanover, New Hampshire, United States of America; 4 Laboratory of Brain Imaging, Neurobiology Center, Nencki Institute of Experimental Biology, Polish Academy of Sciences, Warsaw, Poland; Technion Israel Institute of Technology, ISRAEL

## Abstract

Preferential processing of self-related information is a well-documented phenomenon on both the behavioral and neural levels. However, the impact of self-esteem on this self-preference has not been studied in a systematic way. Here, the electrophysiological correlates of explicit self-reflection were investigated in individuals with low (LSE) and high self-esteem (HSE). Participants evaluated trait adjectives in reference to the self or to an “other” person (close-other, famous) while EEG was recorded. The analysis of event-related potentials focused on the late positive component (LPC), which exhibits a fronto-central distribution and latency over 500 ms. In both LSE and HSE groups, the amplitudes of LPC were enhanced in the self condition when compared to control conditions (both close-other and famous). Crucially, LPC amplitudes in the HSE group were significantly higher than in the LSE group. Moreover, the self-preference effect, defined as the difference between amplitudes of LPC associated with the evaluation of words in relation to oneself vs. other people, was significantly higher in the HSE group than in the LSE group. Overall, our findings indicate that people with high self-esteem tend to engage in self-referential processing to a higher extent.

## Introduction

Self-esteem is one of the oldest and most widely studied constructs in psychology [1, 2]. It refers to the degree to which people value and accept themselves, and reflect their attitude towards their own person [3]. High self-esteem (HSE) implies self-respect, and low self-esteem (LSE) implies self-dissatisfaction or self-contempt [3–5]. Therefore, it might be expected that self-esteem will affect the way people process information about themselves.

Studies on the neural basis of self-referential processing in individuals with LSE and HSE can generally be split into two separate lines of research. The first investigated the processing of self-relevant information in the social functioning context, i.e. in relation to rejection cues and negative feedback from others (e.g. [6–11]). The other group of studies investigated self-referential processing in LSE and HSE groups ‘in isolation’, without any social context, using either an implicit or explicit approach (i.e. either indirectly or directly, respectively). The implicit processing of self-relevant information was assessed [12] while individuals with LSE and HSE completed the implicit association test [13] or while participants were engaged in a task that required a color judgment of personality traits, and the self-relevance of those traits was checked afterwards [14, 15]. However, only two studies have investigated the impact of self-esteem on the neural correlates associated with the process of explicit self-evaluation and self-reflection [16, 17].

First, in a functional magnetic imaging (fMRI) study, participants were asked to evaluate whether a given statement is true of them or of an acquaintance of theirs [16]. A significantly higher activation of the anterior cingulate gyrus, one of the rejection-related neural regions [7], was found in the “self” condition in comparison to the other condition. Crucially, a negative correlation between self-esteem and levels of activation was observed, suggesting that the process of self-evaluation in individuals with LSE relies to a greater extent on mechanisms activated when evaluating-feedback from others. Second, in an event-related potential (ERP) study, individuals with LSE and HSE were required to judge whether personality-trait adjectives accurately described their own person [17]. The recruitment of attentional resources was faster in the LSE group for the processing of highly self-descriptive negative adjectives, as revealed by shorter latencies of the early ERP component P2. This indicates that individuals with LSE pay more attention and are more sensitive to negative information referring to their own person. In both LSE and HSE groups, highly self-descriptive traits evoked enhanced amplitudes of the late positive ERP component (LPC), but LPC amplitude did not differ between groups [17]. However, a main caveat of the Zhang et al.’s [17] study was that a standard control condition (i.e. the other person), commonly used in studies investigating self-referential processing (e.g. [11, 16]), was missing.

The studies by Yang et al. [16] and Zhang et al. [17] suggest that self-esteem modulates brain activity associated with explicit self-reflection. To provide further evidence in support of this notion we investigated the ERP correlates of explicit self-evaluation of personality-trait adjectives in individuals with high and low self-esteem. The control conditions in our experiment involved performing the same task with respect to a close-other and a famous person. While both the close-other and a famous person are familiar to the subject, they differ in respect of their personal relevance to the participants: a famous person is distant and not personally known (not personally relevant), and the close-other is one of the most significant person for the participant (personally relevant). Thus, including both conditions allows addressing whether plausible differences between the self and the other were influenced by the factor of personal relevance.

The self-preference effect has been observed in numerous studies involving different types of stimuli referring to one’s own person (e.g. [18–21]). In the current study, the self-preference effect is operationalized as the difference between amplitudes of ERP component associated with the evaluation of words in reference to oneself and in reference to other people. We focused on LPC, a late positive ERP component with latency over 500 ms and a frontal-central distribution. Specifically, the difference between amplitudes of the LPC, which reflects the different extent to which participants engaged in the reflection process regarding oneself or other people, can be considered as a marker of the preference in this kind of processing. Therefore, a larger difference between the self vs. other condition would imply a higher self-preference effect.

LPC is typically analyzed in studies on self-reflection [17, 21–24], and there is strong evidence indicating significantly enhanced LPC amplitudes when self-related information is processed [21]. We hypothesize that LPC amplitude is modulated by the level of self-esteem, i.e. that individuals with HSE exhibit higher LPC responses to trait adjectives judged as self-referential than individuals with LSE. This supposition is based on Yang et al.’s study [15] on implicit processing of self-related trait adjectives, showing that higher self-esteem level is related to stronger activation of the dorsal medial prefrontal cortex, a brain region highly implicated in self-referential processing [25]. Moreover, we expected that differences in LPC amplitudes between the self and other person conditions (close-other, a famous person) would be more pronounced in individuals with HSE than in individuals with LSE.

## Materials and methods

### Participants

Forty participants were selected based on their self-esteem score, as measured by the Rosenberg questionnaire [3]. This questionnaire assesses a person’s overall evaluation of his or her self-worth. This scale is made up of 10 items such as ‘On the whole, I am satisfied with myself’ or ‘I wish I could have more respect for myself’ and is coded on a 4-point scale ranging from 1 (strongly disagree) to 4 (strongly agree), with the negative items needing to be reverse scored. Based on the Polish standardization of the aforementioned test, participants placed below the 12^th^ percentile were assigned to the low self-esteem group (LSE) and participants placed above the 88^th^ percentile were assigned to the high self-esteem group (HSE). Two participants were excluded from the analysis because of a severe artefact contamination (more than 50% of trials did not pass artefact rejection criteria). Rosenberg scale scores (M ± SD) of the LSE group (n = 18) and HSE group (n = 20) were 21.39 ± 2.03 and 37.70 ± 1.63, respectively. Thirty-five participants were right-handed and three left-handed (as assessed by The Edinburg Inventory [26]). The mean age of the sample was 27.76 ± 3.21 years. All participants reported no history of neurological or psychiatric disorders (subject self-report) and had normal or corrected-to-normal vision.

This study was approved by the local Ethics Committee (University of Social Sciences and Humanities, Warsaw, Poland) and all of the participants gave written informed consent prior to the experiment.

### Stimuli

The set of stimuli consisted of 150 adjectives representing personality traits. The adjectives were adapted from Anderson’s List of Personality-Trait Words in English [27] and translated into Polish. Due to the specific characteristics of the ERP method (segmentation of EEG data typically involves a 1000 ms time-period and is based on the precise timing of stimuli onsets), single words were more suitable for the current study than short sentences [16]. Short sentences–in comparison to single words–require more time to be read, comprehended and assessed in relation to the target of reflection, and that time may substantially vary across participants.

The adjectives were divided into three lists and assigned to three experimental conditions: SELF, CLOSE, and FAMOUS. Each list comprised 50 adjectives (20 positive, 20 negative and 10 neutral), and their emotional content was balanced so that all three had mean emotional values near zero. The list contents were also randomized and balanced with respect to word length (i.e. number of letters) and valence.

### Procedure

Three different conditions were introduced: SELF, CLOSE and FAMOUS. Similarly to our previous studies [28–31], we did not define who should be chosen as a close person to avoid a situation when this particular person (e.g. a specific family member) was not considered close by some participants. The only restriction was that the chosen close person had to have the same gender as the participant. Thus, prior to the experiment, participants were asked to choose a person who was currently (at the time of the experiment) the most significant to them, describe their relationship using The Inclusion of Other in the Self scale (IOS) (M = 5.29; SD = 1.27; min = 3; max = 7), and report on the length of the relationship (M = 18.87; SD = 10.11; min = 3; max = 34). Twenty-two participants chose a family member and sixteen participants chose their friend as the close-other. In the FAMOUS condition, participants were asked to select a famous person from a list of 18 famous Polish actors, actresses, musicians and celebrities (the list contained 9 famous men and 9 famous women). Participants were asked to choose a famous person of the same gender who they were most familiar with. Gender restrictions were introduced in order to avoid the different grammatical forms of adjectives used to describe males and females in Polish.

Trait adjectives were presented centrally on a Flex Scan EV-2450 (Hakusan, Ishikawa, Japan) monitor. Words were displayed with white letters against a black background, with a stimuli size ranging from 3° x 1° to 11° x 1°. Presentation software (Neurobehavioral System, Albany, CA, USA) was used for stimuli presentation and response logging.

Participants were seated in an acoustically shielded dark room at a distance of 50 cm from the screen. The experiment consisted of three blocks (SELF, CLOSE and FAMOUS), the order of which was randomized among subjects. During each block, participants were asked to judge whether a given adjective characterized a person specified in the instruction displayed at the beginning of the block. Subjects responded by pressing one of two buttons on a Cedrus response pad (RB-830, San Pedro, USA), using the index and middle fingers of the right hand to press keys. Key assignment to ‘yes’ and ‘no responses was counterbalanced across subjects.

The experiment started with a detailed instruction explaining the experimental procedure to the participant. Each block was preceded by a short instruction specifying the person whose personal features would be judged in a given block. A single experimental trial consisted of the following sequence of events: white fixation cross presented for 250 ms, black screen visible for 750 ms, an adjective presented for 2000 ms, followed by a black screen displayed for 1500 ms. Participants were asked to respond either when the word was presented or when the black screen was displayed.

### EEG recording

EEG was recorded from 62 electrically shielded scalp electrodes and two additional electrodes placed on the left and right earlobes. A 128-channel amplifier (Quick Amp, Brain Products, Enschede, the Netherlands) and BrainVisionRecorder software (Brain Products, Gilching, Germany) were used to collect EEG signal. Ag/AgCl electrodes were mounted on an elastic cap (ActiCAP, Munich, Germany) according to the extended 10–20 system. Electrode impedances were kept below 5 kΩ. The sampling rate was 500 Hz.

### Behavioural data analysis

The number of yes/no responses and reaction times (RT) were analysed. Responses were scored as yes-responses and no-responses if the appropriate button was pressed within 0–3500 ms after the stimulus onset. If the participant pressed more than one button in a trial, the first response was taken into analyses. RTs were averaged separately across yes-response and no-response trials.

A mixed model repeated measures ANOVA was conducted on the mean number of ‘yes’ responses with ‘condition’ (SELF, CLOSE, FAMOUS) as the within-subject factor and ‘group’ (LSE, HSE) as the between-subject factor.

Mean reaction times (RTs) for yes/no responses were analysed using a mixed model repeated measures ANOVA with within-subject factors of ‘condition’ (SELF, CLOSE, FAMOUS) and ‘type of response’ (yes, no), and with ‘group’ (LSE, HSE) as the between-subject factor. Bonferroni correction for multiple comparisons was applied to the post hoc analyses. The analyses were conducted in IBM SPSS Statistics 21 Advanced Model.

### ERP analysis

The ERP analysis was performed using BrainVisionAnalyzer^®^ software (Brain Products, Gilching, Germany). EEG data were re-referenced to the algebraic average of the signal recorded from the left and right earlobes. This step was done to enable direct comparisons between findings of the current and previously published studies that investigated the self-referential processing, analyzed LPC, and used such reference [12, 17, 21, 23]. Then Butterworth zero phase filters were implemented: high pass– 1 Hz, 12 dB/oct, low pass– 30 Hz, 24 dB/oct, and notch filter– 50 Hz. Correction of ocular artefacts was performed using Independent Component Analysis–ICA. Eye blinks and movements were rejected based on the visual inspection of the components. The EEG data were then segmented into 2 second long epochs with respect to the stimulus onset (-500, 1500 ms). An automatic artefact rejection procedure allowed for maximal permitted amplitudes between -50 and 50 mV. The maximal permitted voltage step per sampling point was 50 μV, and the maximal allowed difference of values in an interval of 200 ms was 100 μV. The lowest allowed activity in an interval of 100 ms was 0.5 μV. Only ‘yes’ responses trials were chosen for further analysis. This was done since confirmatory responses indicated that a given word was suitable for the description of a target of reflection (SELF, CLOSE, FAMOUS). The averaging and baseline correction procedures were applied only to them. This was done separately for each condition. The mean number of segments used to compute ERPs did not differ between conditions: SELF: 26.11 ± 5.05, CLOSE: 21.26 ± 3.92, FAMOUS: 21.95 ± 4.82. In order to visualize the self-preference effect, two differential waves were calculated: SELF-CLOSE and SELF-FAMOUS.

**Fig 1:**
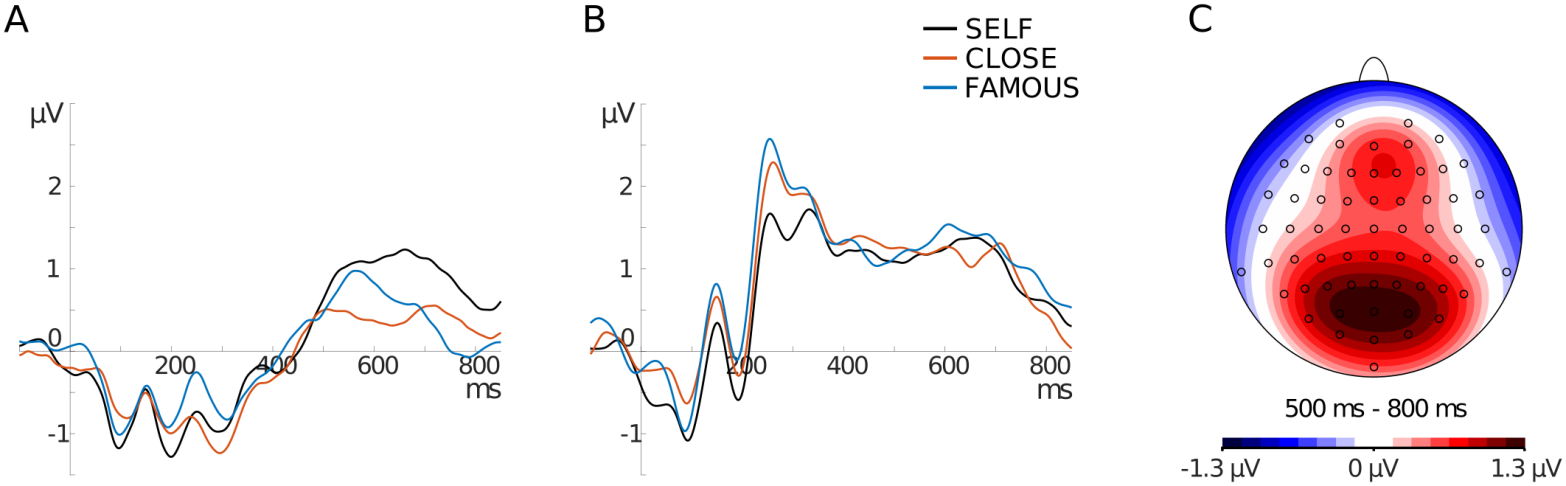
Grand-averaged ERPs for the LSE and HSE groups collapsed together. (A) The frontal region (pooled AFz, Fz, AF4, F2) and (B) parietal region (pooled Pz, POz, PO3, PO4). (C) Topographical distribution of brain activity averaged across the LSE and HSE groups as well as the SELF, CLOSE, and FAMOUS conditions.

Selection of electrodes for analyses has to be orthogonal to potential differences between experimental conditions [32]. Thus, such selection has to be done on the basis of the topographical distribution of brain activity (in the time window corresponding to a given component), averaged across conditions of interest (i.e. LSE and HSE groups; experimental conditions: SELF, CLOSE, FAMOUS). The topographical map of activity, for all experimental conditions and the two groups collapsed together, clearly showed two clusters of activation in the time window corresponding to LPC (i.e. 500–800 ms): a frontal one and a parietal one (see Fig 1C). ERPs at those two regions (averaged across the LSE and HSE groups and 4 electrodes within the maxima of activity) clearly indicated that only at the frontal region, experimental conditions modulated LPC amplitudes (see Fig 1A and 1B). Therefore, LPCs at electrodes within the frontal region were further analyzed.

**Fig 2:**
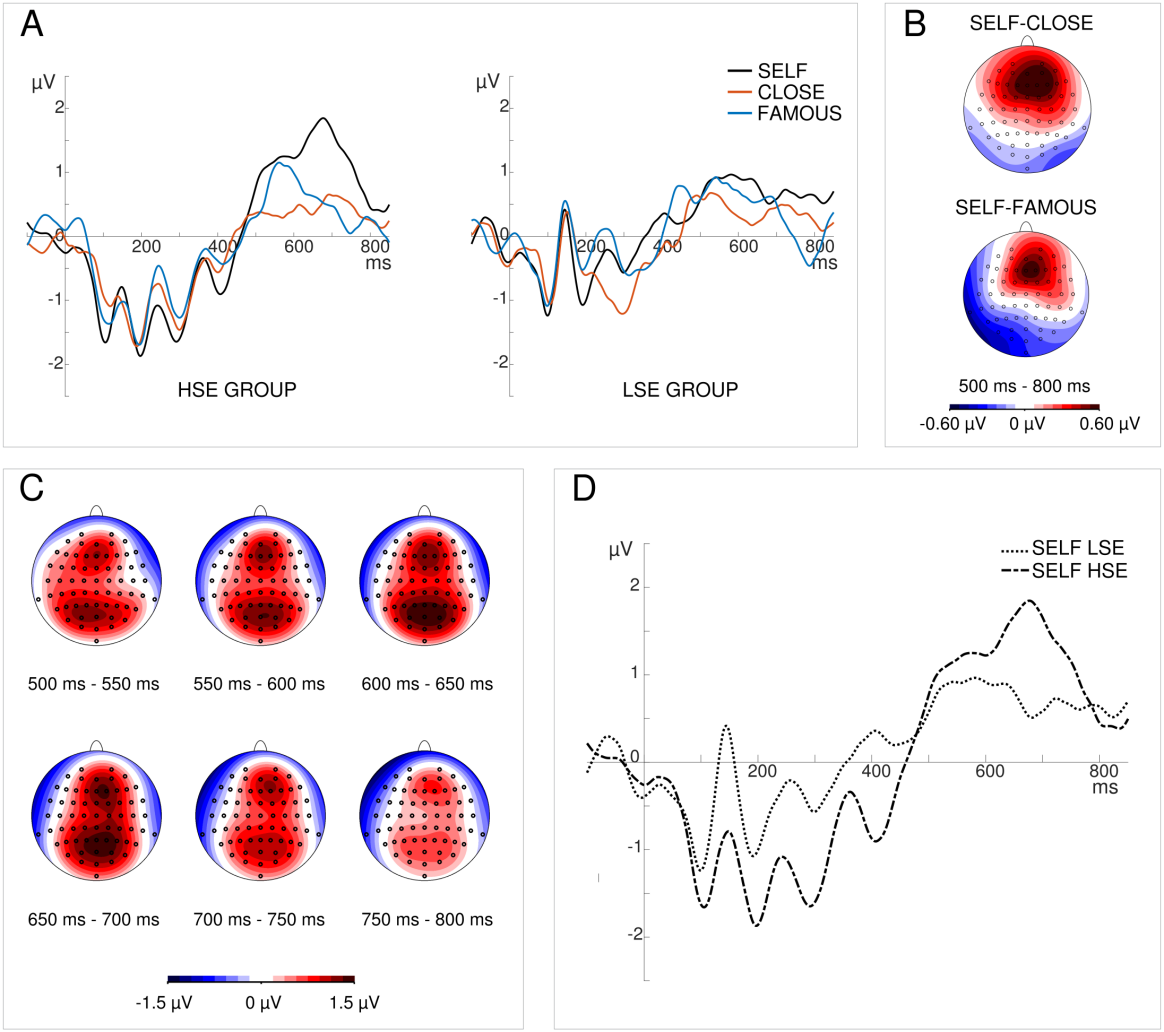
Grand average ERPs and scalp topographies. (A) ERPs in SELF, CLOSE and FAMOUS conditions in HSE (panel A left) and LSE groups (panel A right) pooled over AFz, AF4, Fz, and F2 electrodes. (B) Scalp topographies of difference waves for SELF-CLOSE (upper panel B) and SELF-FAMOUS (lower panel B) conditions for both groups collapsed together. Maximal activity was in the right fronto-central region. (C) Scalp topographies of brain activity recorded in the SELF condition averaged over the LSE and HSE groups. Maximal activity in the region of interest was in the 650–700 ms time window. (D) Grand average ERPs in the SELF condition in both groups. Significant inter-group differences were observed in the 650–700 ms time window.

For analyses, the electrodes AFz, AF4, Fz, and F2 within the frontal region were selected and pooled. This step is justified by the limited spatial resolution of EEG and high correlation between neighboring electrodes. The selection of electrodes was based on two criteria: (1) topographical distribution of brain activity averaged across the LSE and HSE groups as well as the SELF, CLOSE, and FAMOUS conditions (Fig 1C) and (2) the topographical distribution of activity for differential waves, collapsed for two groups together (LSE, HSE) in the 500–800 ms time window (Fig 2B). For each experimental condition, the mean amplitude in the 500–800 ms time window was calculated.

A mixed model repeated measures ANOVA was conducted on LPC amplitudes of ‘yes’ responses with the within-subject factor ‘condition’ (SELF, CLOSE, FAMOUS) and the between-subject factor ‘group’ (LSE, HSE). A similar analysis was performed on the mean amplitudes of differential waves in the 500–800 ms time window. In this case, ANOVA’s within-subject ‘condition’ was defined as SELF-CLOSE and SELF-FAMOUS.

Based on topographical maps of the recorded brain activity distribution in the SELF condition (averaged over LSE and HSE groups), maximal activation was found in the 650–700 ms time window (see Fig 2C). Mean amplitude in this window was calculated for each condition, and repeated measures ANOVA was conducted with the within-subject factor ‘condition’ (SELF, CLOSE, FAMOUS) and between-subject factor ‘group’ (LSE, HSE). A similar analysis was conducted on mean amplitudes of the differential wave in this window, with the within-subject factor ‘condition’ (SELF-CLOSE, SELF-FAMOUS) and between-subject factor ‘group’.

Bonferroni correction for multiple comparisons was applied to all post-hoc analyses. The analyses were conducted in IBM SPSS Statistics 21 Advanced Model.

## Behavioural results

### ‘YES’ responses

**Fig 3:**
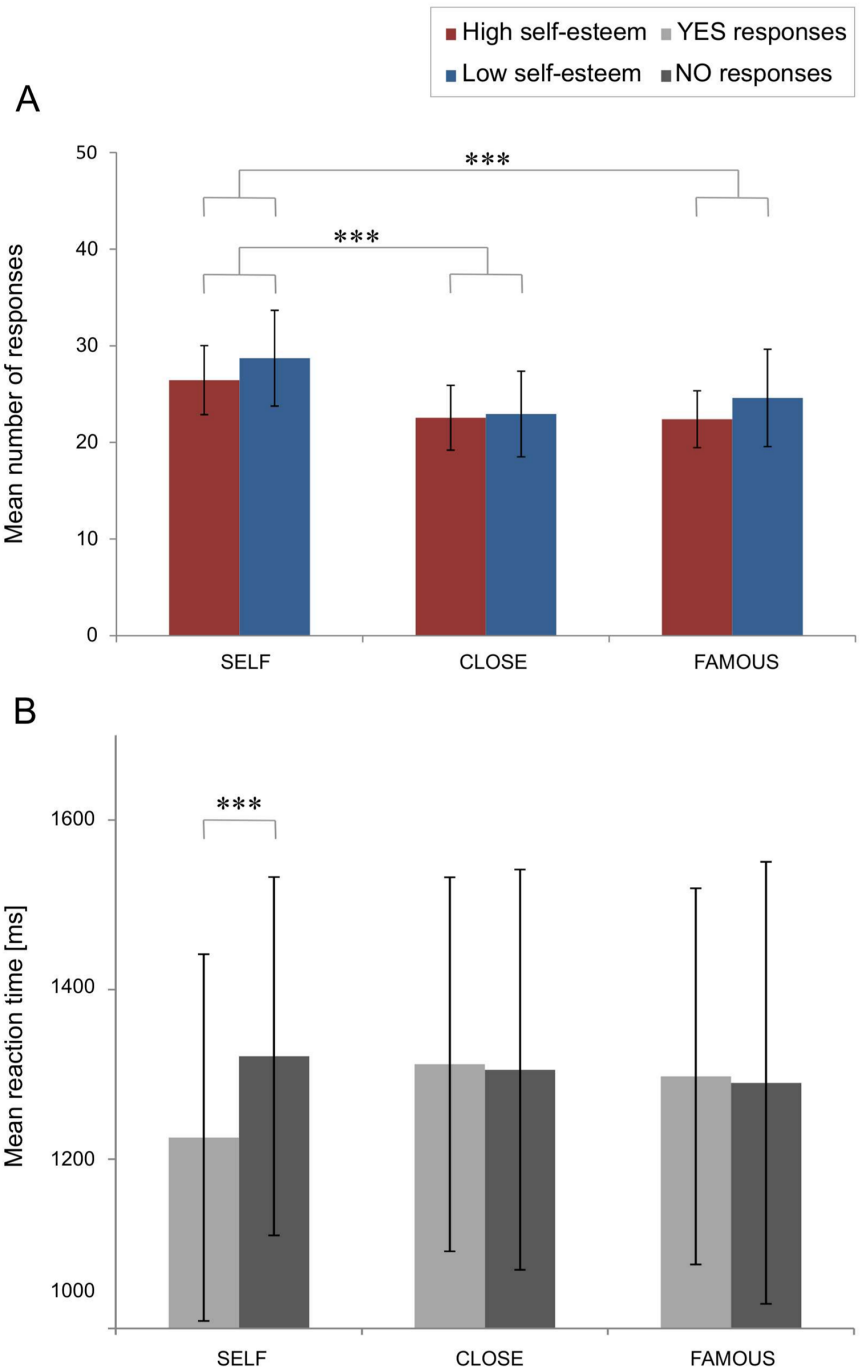
Behavioural results. All data presented as the mean ± standard deviation (SD). (A) The number of YES responses in each group (LSE, HSE) for each experimental condition (SELF, CLOSE, FAMOUS). (B) RTs for ‘yes’ and ‘no’ responses for each condition (SELF, CLOSE, FAMOUS), averaged across the two groups. Significance level is indicated as follows: ***—*p* < 0.001.

The mean numbers of ‘yes’ responses in each condition are presented separately for the LSE and HSE groups in Fig 3A. ANOVA with ‘condition’ as the within-subject factor and ‘group’ as the between-subject factor revealed a significant main effect of ‘condition’ (*F*(2,72) = 19.507; *p* < 0.0001) and the ‘group’ factor reached a trend level (*F*(1,36) = 3.159; *p* = 0.081). Pairwise comparisons yielded significant differences between the number of ‘yes’ responses in the SELF condition vs CLOSE and FAMOUS conditions (*ps* < 0.0001). Participants tended to respond ‘yes’ more often in the SELF condition than for other conditions (SELF: 27.53 ± 4.38; CLOSE: 22.74 ± 3.85; FAMOUS: 23.45 ± 4.17). The ‘condition’ x ‘group’ interaction was non-significant.

### Reaction times

ANOVA conducted on mean RTs with ‘condition’ and ‘type of response’ as within-subject factors and ‘group’ as the between-subject factor yielded two significant interactions: ‘type of response’ x ‘group’ (*F*(1,36) = 7.360; *p* = 0.010) and ‘condition’ x ‘type of response’ (*F*(2,72) = 4.308; *p* = 0.017). Post hoc comparisons for ‘type of response’ x ‘group’ revealed that subjects with LSE had significantly longer RTs for ‘no’ (1349.64 ± 259.89 ms) than for ‘yes’ responses (1269.98 ± 220.73 ms). Post hoc tests for ‘condition’ x ‘type of response’ showed that mean RTs for ‘yes’ and ‘no’ responses in the SELF condition significantly differed–participants tended to answer ‘yes’ faster (see Fig 3B).

## ERPs results

### LPC in 500–800 ms time-window

Grand average ERPs for the LSE and HSE groups are presented in Fig 2A. Repeated measures ANOVA computed on mean amplitudes in the 500–800 ms time-window revealed a significant main effect of ‘condition’: *F*(2,72) = 10.597, *p* < 0.0001. Post hoc comparisons indicated larger amplitudes in the SELF condition than in the CLOSE (*p* < 0.0001) and FAMOUS conditions (*p* = 0.002). The ‘group’ factor was non-significant (*p* = 0.575), suggesting the similar level of activity in both groups. The ‘group’ x ‘condition’ was also non-significant.

**Fig 4:**
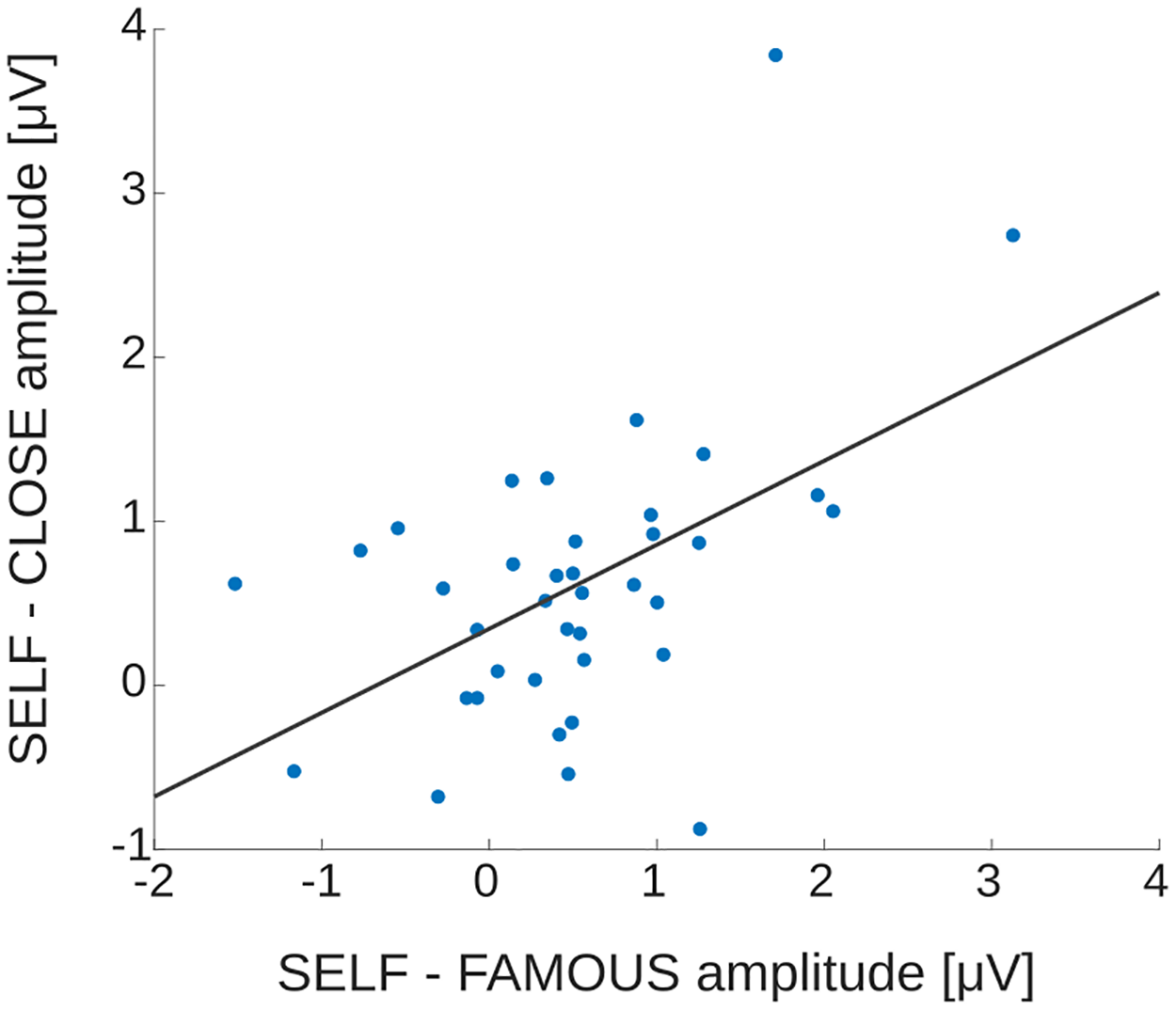
Scatter plot of two measures of the self-preference effect—SELF-FAMOUS and SELF-CLOSE—for all subjects (from both LSE and HSE groups). Data were highly correlated (*r* = 0.508, *p* = 0.001).

ANOVA performed on the mean amplitudes of difference waves showed a trend-level significance of the ‘group’ factor: *F*(1,36) = 3.412; *p* = 0.073 (LSE: 0.335 ± 0.174 μV; HSE: 0.779 ± 0.165 μV). Other effects were non-significant. In addition, the correlation between mean amplitudes of SELF-CLOSE and SELF-FAMOUS difference waves was significant: *r*_*p*_ = 0.508, *p* = 0.001 (see Fig 4).

### LPC in 650–700 ms time-window

Repeated measures ANOVA performed on mean amplitudes in the 650–700 ms time window yielded a significant main effect of only ‘condition’: *F*(2,72) = 4.976, *p* = 0.009. Amplitudes were larger in the SELF condition than in the CLOSE (*p* = 0.009) and FAMOUS conditions (*p* = 0.044). The ‘group’ factor was non-significant (*p* = 0.222). Post hoc comparisons to the significant ‘group’ x ‘condition’ interaction (*F*(2,72) = 3.813, *p* = 0.027) revealed a statistically significant difference between the LSE and HSE groups for the SELF condition (*p* = 0.014), and non-significant differences for other conditions. The HSE group had a larger mean amplitude of LPC in this window than the LSE group (HSE: 1.824 ± 0.336; LSE: 0.560 ± 0.354).

ANOVA computed on mean amplitudes of the difference waves showed a significant main effect of ‘group’: *F*(1,36) = 9.863, *p* = 0.003 (LSE: 0.083 ± 0.280 μV; HSE: 1.294 ± 0.266 μV). The ‘condition’ factor (SELF-CLOSE, SELF-FAMOUS) and its interaction with ‘group’ were non-significant.

## Discussion

In the present ERP study, we investigated the impact of self-esteem on the amplitudes of LPC associated with explicit self-evaluation. We were interested in establishing a plausible link between self-esteem levels and the magnitude of the self-preference effect, defined in the case of our study as the difference between amplitudes of LPC associated with the evaluation of oneself and with evaluation of other people.

On the behavioral level, we found that individuals with LSE required significantly more time to decide that some adjectives were not adequate to characterize a given person (SELF, CLOSE, FAMOUS) in comparison to the time needed to judge them as suitable for the description of that person. In turn, there was no difference between reaction times for “yes” and “no” responses to adjectives in individuals with HSE. However, other effects were common for both groups. Both the LSE and HSE groups more often judged traits as self-descriptive than suitable to describe other people (close-other, famous). Moreover, both groups had significantly shorter RTs to self-descriptive adjectives than to non-self-descriptive adjectives. In general, the latter is in line with the findings of Grundy et al.’s study [12], which showed an analogous effect.

On the neural level, in both the HSE and LSE groups, we observed enhanced LPC amplitudes in the SELF condition in reference to the CLOSE and FAMOUS conditions. It was the case for both (broad, narrow) analyzed time-windows. This finding is in line with numerous studies showing the preferential processing of self-related information (e.g. [33–35]). Specifically, this effect was found for autobiographical information such as the participant’s full name, date of birth, and hometown [36], self-face and self-name [18, 29, 37–41], and self-relevant personality trait words [21, 42, 43]. A converging line of ERP evidence indicates that this self-preference may occur at the early—P200 [40, 42, 43]—and late (P300, LPC) stages of information processing [21, 42, 43]. As far as the impact of self-esteem is concerned, previous studies have shown that self-esteem can modulate the neural correlates of self-referential processing at both stages. For instance, Chen et al. [44] showed larger P200 amplitudes for one’s own name than for a close-other’s name in the LSE group, whereas this P200 effect was not observed in the HSE group. Moreover, in the time window of 300–400 ms after stimulus onset, Grundy et al. showed an inter-group difference in the implicit self-referential processing: larger amplitudes for the me/negative pairing than the me/positive pairing were found only in the HSE group [12].

Another finding of the present study refers to the significant inter-group differences in amplitude of the LPC, which indicates the reflection process [21, 42, 43]. In the SELF condition, mean LPC amplitudes in the narrow time window were substantially higher in individuals with HSE than in individuals with LSE. This result corroborates the findings of an earlier implicit self-evaluation study [16], in which the level of brain activity associated with the processing of self-relevant words correlated positively with self-esteem levels (i.e. higher self-esteem related to higher activation). Similar inter-group effects were also previously observed for other types of self-relevant information. For instance, Oikawa et al. [45] identified the neural correlates of positive evaluation of the self-face and observed that activations of the posterior cingulate cortex (a part of the cortical midline structures associated with self-referential processing) correlated positively with levels of self-esteem.

It is worth noting that previous studies investigating the impact of self-esteem on self-evaluation reported negative findings when analyzing LPC [12, 17]. This discrepancy between the findings of those studies and the current one may be attributed to crucial procedural differences, namely the types of self-referential processing [12] and selection of LSE and HSE groups [17]. In Grundy et al.’s study [12] the process of self-evaluation was assessed in an indirect way, by the strength of association between the self-representation categories and other categories that have a positive or negative valence. Therefore, the findings of that study and of the present study substantially differ with respect to the activated processes: implicit vs. explicit self-evaluation. In Zhang et al.’s [17] study, participants were subdivided into LSE and HSE groups based on a mean split of the Rosenberg self-esteem scale scores (levels of self-esteem in both groups were not provided in their description of experimental procedures). In contrast, we tested individuals with very low (21.39) and very high (37.70) levels of self-esteem that were established on the basis of Rosenberg’s self-esteem scale [3]. We think that this approach unraveled the inter-group differences in LPC responses associated with the explicit evaluation of words referring to personal characteristics.

In the current study the self-preference effect, defined as the difference between amplitudes of LPC associated with the evaluation of words in reference to oneself and in reference to other people, was significantly higher in the HSE group than in the LSE group. However, it was similar for SELF–CLOSE and SELF–FAMOUS conditions in both groups. Therefore, personal relevance did not influence the self-preference effect, probably due to the fact that LPC to FAMOUS and CLOSE conditions did not significantly differ. In fact, both measures of the self-preference effect were highly correlated (see Fig 4). The lack of differences between CLOSE and FAMOUS conditions may suggest that members of both LSE and HSE groups were more focused on thinking about their own person (i.e. they were self-focused), and the process of evaluation in the case of the two control conditions engaged them to a similar extent. This is in contrast to findings of studies, including our own previous investigations, that typically showed the following pattern of ERP results: amplitudes of late ERP components (P300, LPC) were highest in the SELF condition, lowest in the FAMOUS condition, and somewhere in between for the CLOSE condition (e.g. [21, 30, 38, 40]). This discrepancy may be attributed to methodological differences and specific requirements of the current study. However, it should be noted that none of those studies investigated the modulatory effects of self-esteem on self-preferential processing.

Limitations of the current study are as follows. We had a relatively low number of trials; increased number of trials would allow us to compare ERP responses to positive and negative adjectives separately and to investigate the positivity bias, i.e. a pervasive tendency for people to rate positive traits as being truer of themselves than negative traits [46]. Previous studies reported, for instance, that the level of self-esteem was positively correlated with a self-positivity score, i.e. the number of positive words judged as self-relevant and the number of negative words judged as not self-relevant [17]. In a similar vein, people with positive self-views are more likely to show self-positivity than those with negative self-views [47]. Suls et al. [48], however, reported that all people–regardless of their self-esteem level–evaluate positive traits as highly self-descriptive and that only evaluation of negative traits differs with self-esteem. Specifically, as self-esteem level increased, the difference between ratings of positive and negative traits become larger, with high self-esteem participants claiming more positive attributes and fewer negative attributes than did moderate and low self-esteem participants [48]. Thus, with a higher number of trials, it would be possible to view the ERP results in relation to previous studies that focused on negative vs. positive traits evaluated as self-descriptive or not self-descriptive [21, 47, 49]. Moreover, we speculate that the prolonged time of stimuli presentation disturbed the emergence of clear and reliable early ERP components. This is why we did not analyze P200, i.e. an ERP component that was previously reported as being sensitive to self-esteem levels while processing self-referential information [12, 17].

In conclusion, we found that the process of self-evaluation, investigated without any feedback from the external social environment, was associated with substantially increased amplitudes of LPC in individuals with high levels of esteem compared to individuals with low levels of self-esteem. The self-preference effect, i.e. a difference between amplitudes of LPC associated with the evaluation of words in relation to oneself vs. other people (the close-other, a famous person) was not influenced by the personal relevance of the control condition and was also stronger in the HSE group. Therefore, our findings suggest that people with high levels of self-esteem engage to the higher extent in the self-referential processing.

## Supporting information

S1 File Relevant data underlying the findings described in the manuscript. (ZIP)

## References

[1] James W. The principles of psychology (Vol. 1), New York: Dover Publications (original work published in 1890). 1950.

[2] Marsh HW, Scalas LF, Nagengast B. Longitudinal tests of competing factor structures for the Rosenberg Self-Esteem Scale: traits, ephemeral artifacts, and stable response styles. Psychological Assessment 2010 22: 366–381. 10.1037/a0019225 20528064

[3] Rosenberg M. Society and the adolescent self-image. Princeton, NJ: Princeton University Press 1965.

[4] Crocker J, Wolfe CT. Contingencies of self-worth. Psychological Review 2001; 108: 593–623. 10.1037/0033-295x.108.3.593 11488379

[5] Pruessner JC, Baldwin MW, Dedovic K, Renwick R, Mahani NK, Lord C, et al Self-esteem, locus of control, hippocampal volume, and cortisol regulation in young and old adulthood. Neuroimage 2005; 28: 815–826. 10.1016/j.neuroimage.2005.06.014 16023372

[6] Onoda K, Okamoto Y, Nakashima K, Nittono H, Yoshimura S, Yamawaki S, et al Does low self-esteem enhance social pain? The relationship between trait self-esteem and anterior cingulate cortex activation induced by ostracism. Social Cognitive and Affective Neuroscience 2010; 5: 385–391. 10.1093/scan/nsq002 20144945 PMC2999754

[7] Eisenberger NI, Inagaki TK, Muscatell KA, Byrne Haltom KE, Leary MR. The neural sociometer: Brain mechanisms underlying state self-esteem. Journal of Cognitive Neuroscience 2011; 23: 3448–3455. 10.1162/jocn_a_00027 21452934

[8] Li H, Zeigler-Hill V, Luo J, Yang J, Zhang Q. Self-esteem modulates attentional responses to rejection: evidence from event-related brain potentials. Journal of Research in Personality 2012a; 46: 459–464. 10.1016/j.jrp.2012.02.010

[9] Li H, Zeigler-Hill V, Yang J, Jia L, Xiao X, Luo J, et al Low self-esteem and the neural basis of attentional bias for social rejection cues: Evidence from the N2pc ERP component, Personality and Individual Differences 2012b; 53: 947–951. 10.1016/j.paid.2012.03.004

[10] Li H, Yang J. Low self-esteem elicits greater mobilization of attentional resources toward emotional stimuli. Neuroscience Letters 2013; 548: 286–290. 10.1016/j.neulet.2013.05.071 23769724

[11] Guan L, Zhao Y, Wang Y, Chen Y, Yang J. Self-esteem modulates the P3 component in response to the self-face processing after priming with emotional faces. Frontiers in Psychology 2017; 8, 1399, 10.3389/fpsyg.2017.01399 28868041 PMC5563379

[12] Grundy JG, Benarroch MF, Lebarr AN, Shedden JM. Electrophysiological correlates of implicit valenced self-processing in high vs low self-esteem individuals. Social Neuroscience 2015; 10: 100–112. 10.1080/17470919.2014.965339 25265067

[13] Greenwald AG, McGhee DE, Schwartz JLK. Measuring individual differences in implicit cognition: The implicit association test. Journal of Personality and Social Psychology 1998; 74: 1464–1480. 10.1037/0022-3514.74.6.1464 9654756

[14] Yang J, Guan L, Dedovic K, Qi M, Zhang Q. The neural correlates of implicit self-relevant processing in low self-esteem: an ERP study. Brain Research 2012a; 1471: 75–80. 10.1016/j.brainres.2012.06.033 22765911

[15] Yang J, Dedovic K, Guan L, Chen Y, Qi M. Self-esteem modulates dorsal medial prefrontal cortical response to self-positivity bias in implicit self-relevant processing. Social Cognitive and Affective Neuroscience 2014; 9(11): 1814–1818. 10.1093/scan/nst181 24396003 PMC4221225

[16] Yang J, Dedovic K, Chen Y, Zang Q. Self-esteem modulates dorsal anterior cingulate response in self-referential processing. Neuropsychologia 2012b; 50: 1267–1270. 10.1016/j.neuropsychologia.2012.02.010 22391476

[17] Zhang H, Guan LL, Qi MM, Yang J. Self-esteem modulates the time course of self-positivity bias in explicit self-evaluation. PLoS One 2013; 8: e81169 10.1371/journal.pone.0081169 24339908 PMC3855207

[18] Moran JM, Macrae CN, Heatherton TF, Wyland CL, Kelley WM. Neuroanatomical evidence for distinct cognitive and affective components of self. Journal of Cognitive Neuroscience 2006; 18: 1586–1594. 10.1162/jocn.2006.18.9.1586 16989558

[19] Lenggenhager B, Tadi T, Metzinger T, Blanke O. Video ergo sum: manipulating bodily self-consciousness. Science 2007; 317: 1096–1099. 10.1126/science.1143439 17717189

[20] Tacikowski P, Nowicka A. Allocation of attention to self-name and self-face: An ERP study. Biological Psychology 2010; 84: 318–324. 10.1016/j.biopsycho.2010.03.009 20298741

[21] Kotlewska I, Nowicka A. Present-self, past-self and the close-other: neural correlates of assigning trait adjectives to oneself and others. European Journal of Neuroscience 2016; 44: 2064–2071. 10.1111/ejn.13293 27285486

[22] Luo Y, Huang X, Chen Y, Jackson T, Wei D. Negativity bias of the self across time: an event-related potentials study. Neuroscience Letters 2010; 475: 69–73. 10.1016/j.neulet.2010.03.042 20338218

[23] Shestyuk AY, Deldin PJ. Automatic and strategic representation of the self in major depression. American Journal of Psychiatry 2010; 167, 536–544. 10.1176/appi.ajp.2009.06091444 20360316

[24] Fields EC, Kuperberg GR. It’s all about you: An ERP study of emotion and self-relevance in discourse. Neuroimage 2012; 62: 562–574. 10.1016/j.neuroimage.2012.05.003 22584232 PMC3678961

[25] Northoff G, Bermpohl F. Cortical midline structures and the self. Trends in Cognitive Sciences 2004; 8: 102–107. 10.1016/j.tics.2004.01.004 15301749

[26] Oldfield RC. The assessment and analysis of handedness: the Edinburgh inventory. Neuropsychologia 1971; 9: 97–113. 10.1016/0028-3932(71)90067-4 5146491

[27] Anderson NH. Likableness ratings of 555 personality-trait words. Journal of Personality and Social Psychology, 1968; 9, 272–279. 10.1037/h0025907 5666976

[28] Tacikowski P, Brechmann A, Nowicka A. Cross-modal pattern of brain activations associated with the processing of self- and significant other’s name. Human Brain Mapping 2013; 34: 2069–2077. 10.1002/hbm.22048 22431327 PMC6869889

[29] Cygan HB, Tacikowski P, Ostaszewski P, Chojnicka I, Nowicka A. Neural correlates of own name and own face detection in Autism Spectrum Disorder. PLoS One 2014; 9: e86020 10.1371/journal.pone.0086020 24465847 PMC3899112

[30] Kotlewska I, Nowicka A. Present self, past self and close-other: event-related potential study of face and name detection. Biological Psychology 2015; 110: 201–211. 10.1016/j.biopsycho.2015.07.015 26234961

[31] Kotlewska I, Wójcik MJ, Nowicka MM, Marczak K, Nowicka A. Present and past selves: a steady-state visual evoked potentials approach to self-face processing. Scientific Reports 2017; 7(1): 16438 10.1038/s41598-017-16679-6 29180637 PMC5703895

[32] Kriegeskorte N, Simmons WK, Bellgowan PSF, Baker CI. Circular analysis in systems neuroscience–the dangers of double dipping. Nature Neuroscience 2009; 12: 535–540. 10.1038/nn.2303 19396166 PMC2841687

[33] Gray HM, Ambady N, Lowenthal WT, Deldin P. P300 as an index of attention to self-relevant stimuli. Journal of Experimental Social Psychology 2004; 40: 216–224. 10.1016/S0022-1031(03)00092-1

[34] Sedikides C, Gregg AP. Self-enhancement: Food for thought. Perspectives on Psychological Science 2008; 3(2): 102–116. 10.1111/j.1745-6916.2008.00068.x 26158877

[35] Humphreys GW, Sui J. Attentional control and the self: The Self-Attention Network (SAN). Cognitive Neuroscience 2015; 8928: 5–17. 10.1080/17588928.2015.1044427 25945926

[36] Hu X, Wu H, Fu G. Temporal course of executive control when lying about self-and other-referential information: an ERP study. Brain Research 2011; 1369: 149–157. 10.1016/j.brainres.2010.10.106 21059343

[37] Zhao K, Yuan J, Zhong Y, Peng Y, Chen J, Zhou L, et al Event-related potential correlates of the collective self-relevant effect. Neuroscience Letters 2009; 464: 57–61. 10.1016/j.neulet.2009.07.017 19595741

[38] Tacikowski P, Brechmann A, Marchewka A, Jednoróg K, Dobrowolny M, Nowicka A. Is it about the self or the significance? An fMRI study of self-name recognition. Social Neuroscience 2011a; 6: 98–107. 10.1080/17470919.2010.490665 20602286

[39] Tacikowski P, Jednoróg K, Marchewka A, Nowicka A. How multiple repetitions influence the processing of self-, famous and unknown names and faces: an ERP study. International Journal of Psychophysiology 2011b; 79: 219–230. 10.1016/j.ijpsycho.2010.10.010 21035509

[40] Fan W, Chen J, Wang X-Y, Cai R, Tan Q, Chen Y, et al Electrophysiological correlation of the degree of self-reference effect. PLoS One 2013; 8: e80289 10.1371/journal.pone.0080289 24312467 PMC3846566

[41] Tacikowski P, Cygan HB, Nowicka A. Neural correlates of own and close-other’s name recognition: ERP evidence. Frontiers in Human Neuroscience 2014; 8: 194 10.3389/fnhum.2014.00194 24772076 PMC3983482

[42] Mu Y, Han S. Neural oscillations involved in self-referential processing. Neuroimage 2010; 53: 757–768. 10.1016/j.neuroimage.2010.07.008 20633661

[43] Liu Y, Sheng F, Woodcock KA, Han S. Oxytocin effects on neural correlates of self-referential processing. Biological Psychology 2013; 94: 380–387. 23965321 10.1016/j.biopsycho.2013.08.003

[44] Chen J, Shui Q, Zhong Y. Self-esteem modulates automatic attentional responses to self-relevant stimuli: evidence from event-related brain potentials. Frontiers in Human Neuroscience 2015; 9: 376 10.3389/fnhum.2015.00376

[45] Oikawa H, Sugiura M, Sekiguchi A, Tsukiura T, Miyauchi CM, Hashimoto T, et al Self-face evaluation and self-esteem in young females: an fMRI study using contrast effect. Neuroimage 2012; 59: 3668–3676. 10.1016/j.neuroimage.2011.10.098 22079451

[46] Heine SJ, Lehman DR, Markus HR, Kitayama S. Is there a universal need for positive self-regard? Psychological Review 1999; 106: 766–794. 10.1037/0033-295X.106.4.766 10560328

[47] Mezulis AH, Abramson LY, Hyde JS, Hankin BL. Is there a universal positivity bias in attributions? A meta-analytic review of individual, developmental, and cultural differences in the self-serving attributional bias. Psychological Bulletin 2004; 130: 711–747. 10.1037/0033-2909.130.5.711 15367078

[48] Suls J, Lemos K, Stewart HL. Self-esteem, construal, and comparisons with the self, friends, and peers. Journal of Personality and Social Psychology 2002; 82: 252–261. 10.1037//0022-3514.82.2.252 11831414

[49] Brown JD, Brown MA. Self-reflection and feelings of self-worth: When Rosenberg meets Heisenberg. Journal of Experimental Social Psychology 2011; 47: 1269–1275. 10.1016/j.jesp.2011.05.019

